# Supplementary material for: Approach to hyperuniformity in a metallic glass-forming material exhibiting a fragile to strong glass transition
Source: Eur Phys J E Soft Matter. 2023 Jun 28;46(6):50. doi: 10.1140/epje/s10189-023-00308-4 (PMC10307697; doi:10.1140/epje/s10189-023-00308-4)
Supplement: Supplementary file 1 — Supplementary file1 (PDF 376 kb) [file 10189_2023_308_MOESM1_ESM.pdf]

Supplementary Information: Approach to Hyperuniformity in a Metallic Glass-Forming Material Exhibiting a Fragile to Strong Glass Transition

Hao Zhang[1†], Xinyi Wang[1], Jiarui Zhang[1], Hai-Bin Yu[2], Jack F. Douglas[3†]

[1] Department of Chemical and Materials Engineering, University of Alberta, Edmonton, Alberta, Canada, T6G 1H9

[2] Wuhan National High Magnetic Field Center, Huazhong University of Science and Technology, Wuhan, Hubei, China, 430074

[3] Material Measurement Laboratory, Material Science and Engineering Division, National Institute of Standards and Technology, Maryland, USA, 20899

*Corresponding authors: hao.zhang@ualberta.ca; jack.douglas@nist.gov

A. Fragile-Strong Transition, Liquid-liquid Transition, and Equilibrium Polymerization

As we discuss in some detail below, many cooled GF fluids seem to exhibit the formation of linear polymeric clusters whose size grows upon cooling. This situation makes the theory and simulations of Sciortino and coworkers [1] of the molecular clustering of fluid particles forming dynamic linear polymer chains of obvious relevance to modeling the low-q upturn in $S(q)$ of GF liquids and other complex fluids in which this type of dynamic clustering is prevalent. As anticipated, a direct comparison of $S(q)$ data for a simulated fluid undergoing equilibrium polymerization (see Fig. 13 of Sciortino et al.) with light scattering measurements of $S(q)$ on orthoterphenyl, a model GF liquid of the OGF type [2], upon approaching its T_g, reveals a remarkable resemblance, and in many measurements on other GF systems, this type of scattering reveals itself as a low-q upturn with no obvious tendency of the scattering intensity to saturate to finite value at very low q [2-3], presumably due to the large size of the polymeric clusters forming in these systems in comparison to the wavelength of the scattering radiation probing them. This "anomalous" scattering in GF liquids has traditionally been attributed to mysterious "Fisher clusters" [2-3], named in honor of the scientist who most extensively studied this striking phenomenon. Despite its common occurrence, this conspicuous scattering feature of GF liquids is often ignored because of any traditional interpretation of this phenomenon. We suggest that this type of scattering data provides direct evidence about the formation of polymeric clusters in cooled liquids, which notably do not necessarily exist in a state of equilibrium in real cooled liquids because of the extremely long times required for equilibration to occur in such self-assembly processes. It is stressed that this type of structure formation and the corresponding $S(q)$ obtained from such clusters do not give rise to a pre-peak feature in $S(q)$. This scattering feature, which is found in some but not all GF

liquids, is telling us that the structural organization in the liquid is more complex than chain-like structures having a linear topology.

The formation of dynamic polymer chains is just one "universality class" of equilibrium polymerization. More generally, associating molecular and atomic species (specific examples considered below) capable of multi-valent associations normally results in the formation of randomly branched equilibrium polymers in which the polymer size distribution and fractal geometry of the polymeric clusters are *completely different* from the case of linear chain formation at equilibrium. [4-5] This is a physical situation with network-forming GF liquids, such as water and silica, and this type of dynamic self-assembly process occurs in numerous supramolecular processes in aqueous solutions that can potentially inform their counterpart in GF liquids. Finally, for completeness, we mention the third general class of equilibrium self-assembly processes in which the assemblies take the form of compact objects such as spherical micelles and spherical viral capsid structures.

Unfortunately, there is no exact analytic theory of $S(q)$ for fluids exhibiting this common type of supramolecular assembly, but it is possible to investigate this type of self-assembly process by molecular dynamics, Brownian dynamics or Monte Carlo simulation. The scientific literature on $S(q)$ in this type of self-assembling system is extensive, but a full understanding of how to model $S(q)$ does not currently exist. We can nonetheless understand some of the general trends in the scattering data shown in Fig. 1 based on this type of simulation study and from scattering measurements on model randomly branched polymer materials. Most basically, $S(q)$ in this type of branched polymer structure exhibits a low-q upturn as found for linear polymers, but the scattering from these structures often exhibits a pre-peak feature at higher q reflecting the mesh structure of the network or other characteristic dimension of the branched polymer. This type of

structural organization is common in simulations and measurements of $S(q)$ made on polyelectrolyte solutions [6-9] and bottlebrush polymers. [10] The importance of this class of polymer solutions has led to simulations of $S(q)$ of model network polymers [8-9, 11-12] to gain insight into these scattering features in these complex fluids and how these scattering features relate to the molecular geometry of branched polymers.

The pre-peak generally corresponds to some type of mesoscale organization of the polymeric network, but it has been difficult to tie down a unique interpretation that applies to all branched polymeric structures. In the future, we plan to further simulate model networks of different types, formed by self-assembly, or as structures with an assumed static topological structure, to better understand the structural origin of the pre-peak in diverse materials in which randomly branched polymeric structures arise. We next more discuss the nature of equilibrium polymerization in particular GF liquids in a chemically specific way.

There has been intense research recently aimed at quantifying structural organization in metallic GF materials [13-17] and computational and ultrahigh-resolution measurement studies have consistently indicated that the "structuring" in these materials involves a kind of short-range ordering (SRO) at atomic scales in which larger "solute" atoms, such as our Sm atoms in our Al-Sm metallic glass system, are "solvated" by the smaller atomic species to form well-defined clusters in the metallic GF liquid having an approximate local icosahedral symmetry, while the structure formation at larger scales corresponding to "medium range ordering" (MRO) in the material corresponds to *polymeric structures* comprised of the primary icosahedral clusters. The physical situation in metallic GF liquids at low T then resembles the hierarchical assembly of worm-like micelles and amyloid fibers in aqueous solutions in which compact clusters first form, and then these structures self-assemble in turn to form *polymeric assembles* that coexist with the

smaller clusters. [18-23] In particular, both measurements and simulations have revealed in the *strings of icosahedra* form and these structures organize into domains at a larger scale typically on the order of a few nm. These relatively "ordered" regions are surrounded by relatively "loosely-packed" regions [14, 16-17] that are largely "disordered" and lower in density, in addition to cavity regions devoid of particles altogether. Nanoprobe measurements of the local elasticity of metallic glass materials have provided additional insights into this nanoscale heterogeneity. [24-25] These observations are broadly consistent with the occurrence of a kind of microphase separation in which there are coexisting phases of distinct entropy, a phenomenon investigated in many works on metallic and metallic oxide GF materials, [26-28] organic GF liquids [29-31] and discussed intensively recently in many simulations and experimental studies in connection with understanding the thermodynamic and dynamic properties of water at low T. [32-35] We examined this type of structure formation in our earlier work on the Al-Sm metallic glass system. [36-37]

B. Specific Examples of Fluids Exhibiting Equilibrium Polymerization

We note that there is evidence of "string" formation of this kind even in single-component atomic fluids, which likewise exhibit FS glass formation and microphase phase separation. In particular, this type of *equilibrium polymerization* process arises in liquids of chalcogenide elements where there is a propensity for two-fold coordination because of the two "lone pair" electrons of these elements. [38] Equilibrium polymerization has also been suggested in other elements such as C under conditions where sp^3 hybridization of the atomic orbitals is prevalent.[39] The literature is very extensive, and here we simply mention some representative references: S [40-43], P [38, 44], and Se. [45-47] Simulation studies indicate that this phenomenon arises in C [39], Te [48-51], Ge [52], Ga [53-54] and other elements and recent measurements [55] on supercooled liquid Te have

indicated a pre-peak in the structure factor, along with anomalies in its thermodynamic response functions that are similar to those observed in water and our Al-Sm glass-forming liquid.

Although the propensity of the atomic species to form polymeric structures upon cooling is inherently linked to the chemistry of the atomic species in atomic fluids, the formation of polymeric icosahedral clusters is also commonly observed in simulations [56] of hard sphere fluids. Such clusters have apparently been observed in model granular "hard sphere" fluids. [57] Many-body effects can evidently also give rise to this type of self-assembly process due to a purely entropic driving force. Remarkably, this tendency towards anisotropic bonding arises even though the individual particles do not possess any anisotropy in their intermolecular potential.

We note that the tendency of molecules and particles to form dynamic polymeric structures at low T can be modelled roughly by a simple two-state model corresponding to an associated or "polymeric" species and the un-associated or "monomer" species as a coarse-grained model that neglects the polydispersity in this type of dynamic heterogeneity. [58] There is a long history of invoking this type of simplified model to describe the dynamics of water and other network forming liquids, and Tanaka and coworkers [59-62] have developed an interesting variant of this type of model based on the assumption of the dynamic coexistence between water molecules participating in locally energetically preferred structures and water molecules existing in a "normal" liquid-like state. Despite the neglect of the specific structural form of the locally preferred structures, their remarkably simple model is apparently able to capture many aspects of the thermodynamics and dynamics of water, including the "anomalies" in thermodynamic response functions of the type shown in Fig. 3 of our paper and the striking change from a high-T Arrhenius regimes to a low-T Arrhenius regime of diffusion and structural relaxation that is apparently characteristic of liquids exhibiting fragile-to strong glass-formation . Douglas et al. [58] have shown

that the variable cooperativity of equilibrium polymerization transition that arise from *constraining* the chain of particle association events in the polymer formation process can be quantitatively emulated by varying the order *m* of the associative reaction process in the mean two-state model of thus type dynamic structure formation, this type of model having long-standing applications as a simplified model for micelle formation and formation of various biological assemblies. [58] Variable cooperativity in a similar mathematical sense is also basic feature of glass-formation, [63] and we discuss this basic property of thermodynamic polymerization transitions below since it strongly relates to the propensity to whether a fluid exhibits liquid-liquid phase separation. While the simple two-state model is highly convenient for understanding many qualitative aspects of fluids exhibiting dynamic polymeric assembly there are certain phenomena in which the specific geometrical form and structural polydispersity of the "locally energetically preferred structures" discussed by Tanaka and coworkers [59, 62] (dynamic polymers in the language of the present work) can be expected to become of significant importance. We next discuss how the propensity for liquid-liquid phase separation can be understood from an equilibrium polymerization perspective as an example of this structural sensitivity.

The analytic theory of fluids undergoing equilibrium polymerization, in conjunction with phase separation, reveals an interesting (arguably "strange') many-body phenomenon that can arise from the coupling of these distinct thermodynamic transitions which seems to be directly relevant to systems exhibiting FS glass-formation. In particular, when the polymerization transition is highly cooperative in the precise sense of approaching a second-order phase transition due to thermal activation or initiation by a chemical species, [58, 64] the phase boundary can develop *two* critical points, one critical point in the vicinity of the transition near the fluid without the associative interaction giving rise to equilibrium polymerization and a second "liquid-liquid"

critical point at much lower temperatures and at a greatly altered critical concentration corresponding to where the polymerization transition line intersects the phase boundary of the "primary" critical point. We will discuss the polymerization transition line and its possible significance for FS GF liquids below. Interestingly, the lower liquid-liquid critical point involves the coexistence of polymers of different topologies. The equilibrium polymer theory just mentioned refers to the restricted case of the formation of linear equilibrium polymers, but multiple critical points also arise by an apparently similar mechanism in fluids in which randomly branched polymers form by equilibrium polymerization. [65] This is potentially relevant to the current discussion because Naserifar and Goddard [66] have reported based on first-principle quantum calculations that liquid water, a model fluid exhibiting FS glass formation, can be described as "polydisperse branched polymer" material. Recent simulation observations by Sciortino and coworkers [67] have indicated that the high and low density forms of water associated with low-T liquid-liquid phase separation in this fluid corresponds to the hierarchical assembly into tetrahedral water clusters, which in turn self-assemble at a larger scale into polydisperse branched polymers having distinct topological complexity, which is highly reminiscent of observations of hierarchical polymerization of "superstructures" in metallic glasses, mentioned above. We hypothesize based on these theoretical findings and simulation observations that the high fragility of glass-formers at elevated temperatures characteristic of glass-formers exhibiting FS glass-formation should naturally engender a tendency toward liquid-liquid phase transition between self-assembled structures of different topology at lower temperatures as a general attribute of GF liquids exhibiting FS glass-formation. This situation also allows understanding in a unified way why GF liquids exhibiting less cooperativity should not exhibit liquid-liquid phase separation. Betancourt et al.

have suggested a possible physical mechanism modulating the cooperativity of the polymerization transition in GF liquids. [68]

Detailed calculations indicate that the occurrence of *multiple* critical points in systems undergoing *linear chain polymerization* only occurs under a rather special set of conditions in which the polymerization transition is *highly cooperative* and the magnitude of the short-range van der Waals interaction to the interaction strength of the interaction giving rise to the formation of polymeric structures is relatively weak. [64] Water, for example, would seem to satisfy these conditions since this fluid has a relatively high cohesive energy density, and water molecules have relatively large dipolar and quadrupolar interactions that naturally engender a tendency toward directional self-assembly into polymeric structures. Cooperativity in equilibrium polymerization[58] can be quantified similarly to GF liquids in terms of the rate of change of the configurational entropy when T is varied, where this variation in thermodynamics bears a direct relationship to the formation of polymers as a kind of structural "ordering". [63 68-71]

The impact of this type of equilibrium polymerization process on the viscoelastic and stress relation of GF liquids has been emphasized by Douglas and Hubbard, [72] but this theory for relaxation and creep under steady stress remains to be quantitatively tested. This theory predicts many observed aspects of GF liquids such as α-β bifurcation, decoupling, stretched exponential relaxation, Andrade creep, etc. [72-74] Previous measurements have demonstrated the predictive power of standard polymer models for quantitatively understanding the viscoelasticity of cooled liquid metallic materials with a known propensity for equilibrium polymerization (e.g., Se, Te) [75-76], and there would appear to considerable scope for this approach given the ubiquity of the observation of polymerization processes in association with the ordering of metallic glass and other GF materials.

Interestingly, the idea that polymerization underlies glass-formation can be traced back to the advent of the theoretical study of GF liquids. In particular, Hägg [77] in 1934 suggested this hypothesis in response to Zachariasen's introduction of the "random network model" of glasses.[78] He suggested that rather than the atomic positions being truly random, the atoms of both metallic and inorganic glasses form one-dimensional collineations (or two-dimensional sheet polymers or highly perforated sheets, i.e., branched polymers[12, 79]), often composed tetrahedra in ion complexes or arising from the bonding habits of low coordination in "metalloid" elements such as the chalcogenides. In modern terminology, Hägg suggested that the purely random configuration of particles is energetically unstable to adopt local energetically preferred structures in the liquid that cannot be shared by all the particles because of packing frustration so that the material adopts a correlated heterogeneous structure in which string and sheet-like structures arise. The same driving force of energy minimization drives the formation of crystalline materials, but the energy minimization applies to all particles so that the energy of the system is invariant to permuting the particle positions, a basic attribute of crystalline materials. The exchange symmetry is only approximately true for quasi-crystals except for a set of exceptional atomic defect suites that allow for ordered structures having symmetries (e.g., five-fold rotational symmetry) consistent with perfectly periodic crystals. The same situation holds for the formation sheaths of proteins in viruses where the ordered structures lacking a perfect exchange symmetry, as suggested by Crick and Watson, [80] are replaced by "quasi-equivalent" structures [81-84] in which the defects are distributed to minimize the energy of the "crystalline" structure to form structures having otherwise disallowed symmetries. In a sense, quasi-crystals can be viewed as "quasi-equivalent crystals". Hägg also argued that these structures first formed within the liquid state and grew progressively in size upon cooling, thereby frustrating crystallization because of the reduction of particle

mobility of molecular species localized in the clusters and by topological interactions caused by the "muddle of other chains" that further inhibited atomic movement, i.e., entanglement in modern terms. Apart from the prescience of this model of glass-formation, Hägg's model is especially remarkable given that the concept of polymers was hardly accepted at that time. After a heated exchange between Zachariasen and Hägg, [85-86] his model, unfortunately, sank into oblivion until recently when measurements have largely confirmed his conception of a specific form of "dynamic heterogeneity" that is found in many GF liquids (Evidence is discussed below.).

C. Equilibrium Polymerization on Glass-Formation in Multiple Component Materials

This polymerization model of glass-formation, in which the "ordering" process in many GF liquids is viewed as a form of equilibrium polymerization [63, 72], also has strong implications for the miscibility of metal alloys and other multi-component GF liquids, since polymerization inherently alters the miscibility of mixtures and can lead to a multiplicity of critical points arising from competition of the polymerization thermodynamics and phase separation. [64, 87-88] This issue provides an added level of complexity to metallic GF alloys since the liquid-liquid phase separation between liquid structures having different topologies, as discussed above, should occur simultaneously with ordinary liquid-liquid phase separation between different chemical species. A change in miscibility arising from equilibrium polymerization can be particularly significant when one of the components self-assembles upon heating, as in the case of S. This type of additive is predicted [89] to give rise to closed loop phase behavior, as observed in S-Te mixtures. [90-93]

Tsuchiya [87] explored the relevance of this type of "ordering process" on the thermodynamic stability of Te alloys and he also mentions[87] this phenomenon in S solutions [41] (See Fig. 1a of Ref. 41) and in He^3-He^4 mixtures [94-95]. Note that the liquid-liquid transition between the normal and superfluid liquid states occurs as a line of second-order phase transitions emanating from the

critical point in the He mixtures (a tricritical point). This transition can also be viewed as a polymerization transition line based on Feynman's equilibrium polymerization model of the superfluid transition where the polymers were assumed to involve the collective motion of atoms moving permutationally in the form of ring polymers. [96-97] In our previous work on the Al-Sm metallic glass [36-37], we observed a similar permutational motion in our classical molecular dynamics of the Al-Sm GF liquid, although these dynamic polymer chains (which we call "stings") were not found to be generally closed to form rings. [36] Moreover, these dynamic polymeric structures were shown to be relevant to understanding the *T* dependence of mass diffusion and the relaxation times of this metallic GF liquid.[98]

The phase behavior of associative fluids often involves a line of polymerization or self-assembly transitions that terminate near the phase boundary for liquid-liquid phase separation, giving rise to a tricritical point when the line of transitions intersects the phase boundary at the critical point and a critical endpoint when the polymerization line intersects the binodal of the phase boundary away from the critical point. Importantly, this phenomenon can arise both when the polymerization process involves linear chains [64, 99] and when the polymerization process involves randomly branched polymeric structures. [65] This phenomenon is ubiquitous in complex fluids in which molecular assembly occurs, including micelle formation [100-101], living polymers [41], thermally reversible gelation of polymers in solution [102-103], etc. Liquid-liquid thermodynamic transitions, accompanied by significant changes in fluid structure and viscoelastic properties in the two liquid states, are common in complex fluids, even if this phenomenon is not always recognized. Tanaka has recently provided an up-to-date review of the exotic phenomenon of liquid-liquid phase separation in single component fluids.[104]

D. Suggested Physical Origin of the Widom Line

One of the important features of liquid-liquid phase transitions in fluids exhibiting equilibrium polymerization is that the transition from a monomeric to a viscous polymeric liquid may occur as a rounded second-order thermodynamic transition upon crossing the polymerization line in the one-phase region can be first-order under conditions in which the polymerization line intersects the phase boundary [58, 105] (See Fig. 1 of Ref. 99). The polymerization line often extends beyond the phase boundary, forming an "extension line" into the one-phase region at which the correlation length for composition fluctuations peaks, reflecting the clustering of fluid molecules near the polymerization line. [58, 99] In particular, the polymeric particle clusters become large upon approaching the polymerization line and the scattering function takes the form appropriate for Ornstein-Zernike form for flexible polymers where the correlation length average polymer size grows in parallel. [105]

The "Widom line" [106-107] is often identified in the liquid state literature with a locus in the temperature-density plane at which the correlation length and scattering intensity of a liquid exhibit maximum in the one-phase region, forming a line in this parameter space that typically intersects the phase boundary in the vicinity of the critical point, at which these basic scattering properties diverge. In practice, however, the Widom line is identified in studies of the water, and other liquids thought to exhibit liquid-liquid phase separation, [106, 108] by a characteristic temperature at which C_p exhibits a maximum where a line of such characteristic T is obtained by varying the density through changes in the applied pressure. Liquid-liquid transitions are defined by a peak in C_p in fluids exhibiting supramolecular polymerization. This phenomenon is well exemplified in the Stockmayer fluid [109-110], a simplified model of water that includes a Lennard-Jones interaction in combination with a superimposed dipolar interaction that gives rise to chain assembly. The

appearance of a "ridge" in the supercritical region has been observed since the 1930s when some type of transition between the gas-like and liquid-like organization and molecular clustering was inferred by x-ray scattering, sound propagation and other measurements and this type of observation was organized and emphasized by Nishikawa and coworkers. [111-114] Simulations have recently indicated that this line of transitions does not exist in mixtures of binary Lennard-Jones fluids [115] so the directional interaction is required for self-assembly and the observation of the transition line. We suggest that the extension line of supercooled liquids may be identified with a polymerization line so that the termination of this transition is appropriately made from a maximum in C_p, the condition utilized by Stanley and coworkers for experimentally locating the Widom line. The existence of a polymerization transition in cooled liquids offers a potentially new interpretation of the Widom line.

E. Relation Between FS Glass-Formers and Superionic Crystalline Materials?

Angell [116-117] has suggested that GF liquids undergoing an FS-type transition, including water, silica, BeF_2, and some metallic GF materials, are in some ways "crystal-like in character while remaining strictly aperiodic", and he further suggested that these materials are somehow intermediate between quasi-crystals and ordinary GF liquids. We interpret these impressionistic thoughts as being consistent with effectively hyperuniform materials which are "crystal-like" specifically because of their relatively low values of H. [118-119] Based on this intuitive conception of these materials, he introduced a "bond lattice" model [117, 120-121] to rationalize both thermodynamic and relaxation properties of these odd GF liquids having little or no evidence of a peak in the specific heat as the material goes out of equilibrium, a defining feature of the glass-transition in many experimental studies of conventional materials exhibiting glass-formation, while these materials are also peculiar in that they exhibit a thermodynamic "lambda transition",

characterized by a peak in the specific heat resembling a thermodynamic melting transition, as found in crystalline materials. This is another way these materials are "crystal-like" that his model addresses. He went on to speculate that "plastic crystals" exhibiting orientational disorder and superionic crystalline materials and globular proteins might belong to this same general class of materials. In accord with these remarkable suggestions, we have observed striking similarities between the dynamics of our Al-Sm metallic glass and simulations of the dynamics of superionic UO_2. In our previous simulations of UO_2, we noticed that the *T*-dependent dynamics of this material has a strong resemblance to a singular *T*-dependence of the rate of certain enzyme reactions. [122] Angell's ideas regarding the FS thus seem to have a lot of merits, of course, he does not offer any clear underlying physical reason for this distinct type of glass-formation that might allow some understanding of why all these materials are "related".

References

1. Sciortino, F.; Bianchi, E.; Douglas, J. F.; Tartaglia, P., Self-Assembly of Patchy Particles into Polymer Chains: A Parameter-Free Comparison between Wertheim Theory and Monte Carlo Simulation. *Journal of Chemical Physics* **2007**, *126*, 194903.
2. Patkowski, A.; Thurn-Albrecht, T.; Banachowicz, E.; Steffen, W.; Bosecke, P.; Narayanan, T.; Fischer, E. W., Long-Range Density Fluctuations in Orthoterphenyl as Studied by Means of Ultrasmall-Angle X-Ray Scattering. *Phys. Rev. E* **2000**, *61*, 6909-6913.
3. Fischer, E. W., Light-Scattering and Dielectric Studies on Glass-Forming Liquids. *Physica A* **1993**, *201*, 183-206.
4. Audus, D. J.; Starr, F. W.; Douglas, J. F., Coupling of Isotropic and Directional Interactions and Its Effect on Phase Separation and Self-Assembly. *Journal of Chemical Physics* **2016**, *144*, 074901.
5. Audus, D. J.; Starr, F. W.; Douglas, J. F., Valence, Loop Formation and Universality in Self-Assembling Patchy Particles. *Soft Matter* **2018**, *14*, 1622-1630.
6. Chremos, A.; Douglas, J. F., Communication: Counter-Ion Solvation and Anomalous Low-Angle Scattering in Salt-Free Polyelectrolyte Solutions. *Journal of Chemical Physics* **2017**, *147*, 241103.
7. Chremos, A.; Douglas, J. F., Polyelectrolyte Association and Solvation. *Journal of Chemical Physics* **2018**, *149*, 163305.
8. Horkay, F.; Chremos, A.; Douglas, J. F.; Jones, R. L.; Lou, J. Z.; Xia, Y., Systematic Investigation of Synthetic Polyelectrolyte Bottlebrush Solutions by Neutron and Dynamic Light Scattering, Osmometry, and Molecular Dynamics Simulation. *Journal of Chemical Physics* **2020**, *152*, 194904.
9. Horkay, F.; Chremos, A.; Douglas, J. F.; Jones, R.; Lou, J. Z.; Xia, Y., Comparative Experimental and Computational Study of Synthetic and Natural Bottlebrush Polyelectrolyte Solutions. *Journal of Chemical Physics* **2021**, *155*, 074901.
10. Sarapas, J. M.; Martin, T. B.; Chremos, A.; Douglas, J. F.; Beers, K. L., Bottlebrush Polymers in the Melt and Polyelectrolytes in Solution Share Common Structural Features. *P Natl Acad Sci USA* **2020**, *117*, 5168-5175.
11. Chremos, A.; Horkay, F.; Douglas, J. F., Structure and Conformational Properties of Ideal Nanogel Particles in Athermal Solutions. *Journal of Chemical Physics* **2021**, *155*, 134905.
12. Chremos, A.; Douglas, J. F.; Basser, P. J.; Horkay, F., Molecular Dynamics Study of the Swelling and Osmotic Properties of Compact Nanogel Particles. *Soft Matter* **2022**, *18*, 6278-6290.
13. Sheng, H. W.; Luo, W. K.; Alamgir, F. M.; Bai, J. M.; Ma, E., Atomic Packing and Short-to-Medium-Range Order in Metallic Glasses. *Nature* **2006**, *439*, 419-425.
14. Li, M.; Wang, C. Z.; Hao, S. G.; Kramer, M. J.; Ho, K. M., Structural Heterogeneity and Medium-Range Order in Zrxcu100-X Metallic Glasses. *Phys. Rev. B* **2009**, *80*, 184201.
15. Yavari, A. R., Materials Science - a New Order for Metallic Glasses. *Nature* **2006**, *439*, 405-406.
16. Hirata, A.; Guan, P. F.; Fujita, T.; Hirotsu, Y.; Inoue, A.; Yavari, A. R.; Sakurai, T.; Chen, M. W., Direct Observation of Local Atomic Order in a Metallic Glass. *Nat Mater* **2011**, *10*, 28-33.
17. Zhu, F.; Hirata, A.; Liu, P.; Song, S. X.; Tian, Y.; Han, J. H.; Fujita, T.; Chen, M. W., Correlation between Local Structure Order and Spatial Heterogeneity in a Metallic Glass. *Phys. Rev. Lett.* **2017**, *119*, 215501.
18. Jensen, G. V.; Lund, R.; Gummel, J.; Narayanan, T.; Pedersen, J. S., Monitoring the Transition from Spherical to Polymer-Like Surfactant Micelles Using Small-Angle X-Ray Scattering. *Angew. Chem.-Int. Edit.* **2014**, *53*, 11524-11528.
19. Sun, P. P.; Lu, F.; Wu, A. L.; Shi, L. J.; Zheng, L. Q., Spontaneous Wormlike Micelles Formed in a Single-Tailed Zwitterionic Surface-Active Ionic Liquid Aqueous Solution. *Soft Matter* **2017**, *13*, 2543-2548.
20. Schurtenberger, P.; Cavaco, C.; Tiberg, F.; Regev, O., Enormous Concentration-Induced Growth of Polymer-Like Micelles. *Langmuir* **1996**, *12*, 2894-2899.

21. Jain, S.; Udgaonkar, J. B., Evidence for Stepwise Formation of Amyloid Fibrils by the Mouse Prion Protein. *J Mol Biol* **2008**, *382*, 1228-1241.
22. Dudowicz, J.; Freed, K. F.; Douglas, J. F., Lattice Model of Living Polymerization. Iii. Evidence for Particle Clustering from Phase Separation Properties and "Rounding" of the Dynamical Clustering Transition. *Journal of Chemical Physics* **2000**, *113*, 434-446.
23. Ross, C. A.; Poirier, M. A., Protein Aggregation and Neurodegenerative Disease. *Nat. Med.* **2004**, *10*, S10-S17.
24. Liu, Y. H.; Wang, D.; Nakajima, K.; Zhang, W.; Hirata, A.; Nishi, T.; Inoue, A.; Chen, M. W., Characterization of Nanoscale Mechanical Heterogeneity in a Metallic Glass by Dynamic Force Microscopy. *Phys. Rev. Lett.* **2011**, *106*, 125504.
25. Liu, C. Y.; Maass, R., Elastic Fluctuations and Structural Heterogeneities in Metallic Glasses. *Advanced Functional Materials* **2018**, *28*, 1800388.
26. Aasland, S.; McMillan, P. F., Density-Driven Liquid–Liquid Phase Separation in the System Ai2o3–Y2o3. *Nature* **1994**, *369*, 633-636.
27. Wei, S.; Yang, F.; Bednarcik, J.; Kaban, I.; Shuleshova, O.; Meyer, A.; Busch, R., Liquid-Liquid Transition in a Strong Bulk Metallic Glass-Forming Liquid. *Nat. Commun.* **2013**, *4*, 2083.
28. Shen, J., et al., Metallic Glacial Glass Formation by a First-Order Liquid-Liquid Transition. *J. Phys. Chem. Lett.* **2020**, *11*, 6718-6723.
29. Tanaka, H.; Kurita, R.; Mataki, H., Liquid-Liquid Transition in the Molecular Liquid Triphenyl Phosphite. *Phys. Rev. Lett.* **2004**, *92*, 025701.
30. Zhu, M.; Wang, J. Q.; Perepezko, J. H.; Yu, L., Possible Existence of Two Amorphous Phases of D-Mannitol Related by a First-Order Transition. *Journal of Chemical Physics* **2015**, *142*, 244504.
31. Kobayashi, M.; Tanaka, H., The Reversibility and First-Order Nature of Liquid-Liquid Transition in a Molecular Liquid. *Nat. Commun.* **2016**, *7*, 13438.
32. Poole, P. H.; Grande, T.; Angell, C. A.; McMillan, P. F., Polymorphic Phase Transitions in Liquids and Glasses. *Science* **1997**, *275*, 322-323.
33. Mishima, O.; Stanley, H. E., The Relationship between Liquid, Supercooled and Glassy Water. *Nature* **1998**, *396*, 329-335.
34. Debenedetti, P. G.; Sciortino, F.; Zerze, G. H., Second Critical Point in Two Realistic Models of Water. *Science* **2020**, *369*, 289.
35. Kringle, L.; Thornley, W. A.; Kay, B. D.; Kimmel, G. A., Reversible Structural Transformations in Supercooled Liquid Water from 135 to 245 K. *Science (New York, N.Y.)* **2020**, *369*, 1490-1492.
36. Zhang, H.; Wang, X. Y.; Yu, H. B.; Douglas, J. F., Dynamic Heterogeneity, Cooperative Motion, and Johari-Goldstein Beta-Relaxation in a Metallic Glass-Forming Material Exhibiting a Fragile-to-Strong Transition. *Eur Phys J E* **2021**, *44*.
37. Zhang, H.; Wang, X. Y.; Yu, H. B.; Douglas, J. F., Fast Dynamics in a Model Metallic Glass-Forming Material *Journal of Chemical Physics* **2021**, *154*, 084505.
38. Katayama, Y.; Mizutani, T.; Utsumi, W.; Shimomura, O.; Yamakata, M.; Funakoshi, K., A First-Order Liquid-Liquid Phase Transition in Phosphorus. *Nature* **2000**, *403*, 170-173.
39. Glosli, J. N.; Ree, F. H., Liquid-Liquid Phase Transformation in Carbon. *Phys. Rev. Lett.* **1999**, *82*, 4659-4662.
40. Tobolsky, A. V.; Eisenberg, A., Equilibrium Polymerization of Sulfur. *J Am Chem Soc* **1959**, *81*, 780-782.
41. Greer, S. C., Physical Chemistry of Equilibrium Polymerization. *J Phys Chem B* **1998**, *102*, 5413-5422.
42. Zhang, L. J.; Ren, Y.; Liu, X. R.; Han, F.; Evans-Lutterodt, K.; Wang, H. Y.; He, Y. L.; Wang, J. L.; Zhao, Y.; Yang, W. G., Chain Breakage in the Supercooled Liquid - Liquid Transition and Re-Entry of the Lambda-Transition in Sulfur. *Sci Rep-Uk* **2018**, *8*, 4558.
43. Henry, L.; Mezouar, M.; Garbarino, G.; Sifre, D.; Weck, G.; Datchi, F., Liquid-Liquid Transition and Critical Point in Sulfur. *Nature* **2020**, *584*, 382.

44. Hohl, D.; Jones, R. O., Polymerization in Liquid Phosphorus: Simulation of a Phase Transition. *Phys. Rev. B* **1994**, *50*, 17047-17053.
45. Bohmer, R.; Angell, C. A., Elastic and Viscoelastic Properties of Amorphous Selenium and Identification of the Phase Transition between Ring and Chain Structures. *Phys. Rev. B* **1993**, *48*, 5857-5864.
46. Bichara, C.; Pellegatti, A.; Gaspard, J. P., Chain Structure of Liquid Selenium Investigated by a Tight-Binding Monte Carlo Simulation. *Phys. Rev. B* **1994**, *49*, 6581-6586.
47. Kalikka, J.; Akola, J.; Jones, R. O.; Schober, H. R., Density Functional and Classical Simulations of Liquid and Glassy Selenium. *Phys. Rev. B* **2020**, *102*, 104202.
48. Tsuchiya, Y., Sound Velocity in the Liquid Tl-Se System. *J Non-Cryst Solids* **1993**, *156*, 700-703.
49. Tsuchiya, Y., Thermodynamics of the Structural Changes in the Liquid Ge-Te System around the Te-Rich Eutectic Composition. *J Non-Cryst Solids* **2002**, *312-14*, 212-216.
50. Akola, J.; Jones, R. O.; Kohara, S.; Usuki, T.; Bychkov, E., Density Variations in Liquid Tellurium: Roles of Rings, Chains, and Cavities. *Phys. Rev. B* **2010**, *81*, 094202.
51. Akola, J.; Jones, R. O., Structure and Dynamics in Amorphous Tellurium and Te-N Clusters: A Density Functional Study. *Phys. Rev. B* **2012**, *85*, 134103.
52. Sosso, G. C.; Colombo, J.; Behler, J.; Del Gado, E.; Bernasconi, M., Dynamical Heterogeneity in the Supercooled Liquid State of the Phase Change Material Gete. *J Phys Chem B* **2014**, *118*, 13621-13628.
53. Jara, D. A. C.; Michelon, M. F.; Antonelli, A.; de Koning, M., Theoretical Evidence for a First-Order Liquid-Liquid Phase Transition in Gallium. *Journal of Chemical Physics* **2009**, *130*, 221101.
54. Li, R. Z.; Sun, G.; Xu, L. M., Anomalous Properties and the Liquid-Liquid Phase Transition in Gallium. *Journal of Chemical Physics* **2016**, *145*, 054506.
55. Sun, P., et al., Structural Changes across Thermodynamic Maxima in Supercooled Liquid Tellurium: A Water-Like Scenario. *P Natl Acad Sci USA* **2022**, *119*, e2202044119.
56. Anikeenko, A. V.; Medvedev, N. N., Polytetrahedral Nature of the Dense Disordered Packings of Hard Spheres. *Phys. Rev. Lett.* **2007**, *98*, 235504.
57. Xia, C. J.; Li, J. D.; Cao, Y. X.; Kou, B. Q.; Xiao, X. H.; Fezzaa, K.; Xiao, T. Q.; Wang, Y. J., The Structural Origin of the Hard-Sphere Glass Transition in Granular Packing. *Nat. Commun.* **2015**, *6*, 8409.
58. Douglas, J. F.; Dudowicz, J.; Freed, K. F., Lattice Model of Equilibrium Polymerization. Vii. Understanding the Role of "Cooperativity" in Self-Assembly. *Journal of Chemical Physics* **2008**, *128*, 224901.
59. Shi, R.; Russo, J.; Tanaka, H., Common Microscopic Structural Origin for Water's Thermodynamic and Dynamic Anomalies. *Journal of Chemical Physics* **2018**, *149*, 224502.
60. Shi, R.; Russo, J.; Tanaka, H., Origin of the Emergent Fragile-to-Strong Transition in Supercooled Water. *P Natl Acad Sci USA* **2018**, *115*, 9444-9449.
61. Shi, R.; Tanaka, H., The Anomalies and Criticality of Liquid Water. *P Natl Acad Sci USA* **2020**, *117*, 26591-26599.
62. Yu, Z. H.; Shi, R.; Tanaka, H., A Unified Description of the Liquid Structure, Static and Dynamic Anomalies, and Criticality of Tip4p/2005 Water by a Hierarchical Two-State Model. *J Phys Chem B* **2023**, *127*, 3452-3462.
63. Douglas, J. F.; Dudowicz, J.; Freed, K. F., Does Equilibrium Polymerization Describe the Dynamic Heterogeneity of Glass-Forming Liquids? *Journal of Chemical Physics* **2006**, *125*, 144907.
64. Dudowicz, J.; Freed, K. F.; Douglas, J. F., Lattice Model of Equilibrium Polymerization. Iv. Influence of Activation, Chemical Initiation, Chain Scission and Fusion, and Chain Stiffness on Polymerization and Phase Separation. *Journal of Chemical Physics* **2003**, *119*, 12645-12666.
65. Tanaka, F.; Matsuyama, A., Tricriticality in Thermoreversible Gels. *Phys. Rev. Lett.* **1989**, *62*, 2759-2762.
66. Naserifar, S.; Goddard, W. A., Liquid Water Is a Dynamic Polydisperse Branched Polymer. *P Natl Acad Sci USA* **2019**, *116*, 1998-2003.

67. Neophytou, A.; Chakrabarti, D.; Sciortino, F., Topological Nature of the Liquid-Liquid Phase Transition in Tetrahedral Liquids. *Nature Physics, in press* **2022**, *18*, 1248-1253.
68. Betancourt, B. A. P.; Douglas, J. F.; Starr, F. W., String Model for the Dynamics of Glass-Forming Liquids. *Journal of Chemical Physics* **2014**, *140*, 204509.
69. Starr, F. W.; Douglas, J. F., Modifying Fragility and Collective Motion in Polymer Melts with Nanoparticles. *Phys. Rev. Lett.* **2011**, *106*, 115702.
70. Xu, W. S.; Douglas, J. F.; Xia, W. J.; Xu, X. L., Investigation of the Temperature Dependence of Activation Volume in Glass-Forming Polymer Melts under Variable Pressure Conditions. *Macromolecules* **2020**, *53*, 6828-6841.
71. Xu, W. S.; Douglas, J. F.; Xia, W. J.; Xu, X. L., Understanding Activation Volume in Glass-Forming Polymer Melts Via Generalized Entropy Theory. *Macromolecules* **2020**, *53*, 7239-7252.
72. Douglas, J. F.; Hubbard, J. B., Semiempirical Theory of Relaxation - Concentrated Polymer-Solution Dynamics. *Macromolecules* **1991**, *24*, 3163-3177.
73. Stukalin, E. B.; Douglas, J. F.; Freed, K. F., Multistep Relaxation in Equilibrium Polymer Solutions: A Minimal Model of Relaxation in "Complex" Fluids. *Journal of Chemical Physics* **2008**, *129*, 094901.
74. Douglas, J. F., Integral Equation Approach to Condensed Matter Relaxation. *Journal of Physics-Condensed Matter* **1999**, *11*, A329-A340.
75. Bernatz, K. M.; Echeverria, I.; Simon, S. L.; Plazek, D. J., Characterization of the Molecular Structure of Amorphous Selenium Using Recoverable Creep Compliance Measurements. *J Non-Cryst Solids* **2002**, *307*, 790-801.
76. Faivre, G.; Gardissat, J. L., Viscoelastic Properties and Molecular Structure of Amorphous Selenium. *Macromolecules* **1986**, *19*, 1988-1996.
77. Hagg, G., The Vitreous State. *Journal of Chemical Physics* **1935**, *3*, 42-49.
78. Zachariasen, W. H., The Atomic Arrangement in Glass. *J Am Chem Soc* **1932**, *54*, 3841-3851.
79. Douglas, J. F., Swelling and Growth of Polymers, Membranes, and Sponges. *Phys. Rev. E* **1996**, *54*, 2677-2689.
80. Crick, F. H. C.; Watson, J. D., Structure of Small Viruses. *Nature* **1956**, *177*, 473-475.
81. Caspar, D. L. D.; Klug, A., Physical Principles in the Construction of Regular Viruses. *Cold Spring Harbor Symposia on Quantitative Biology* **1962**, *27*, 1-24.
82. Klug, A., Architectural Design of Spherical Viruses. *Nature* **1983**, *303*, 378-379.
83. Caspar, D. L. D.; Fontano, E., Five-Fold Symmetry in Crystalline Quasicrystal Lattices. *P Natl Acad Sci USA* **1996**, *93*, 14271-14278.
84. Klug, A., Macromolecular Order in Biology. *Philos T R Soc A* **1994**, *348*, 167-178.
85. Zachariasen, W. H., The Vitreous State. *Journal of Chemical Physics* **1935**, *3*, 162-163.
86. Hagg, G., The Vitreous State. *Journal of Chemical Physics* **1935**, *3*, 363-364.
87. Tsuchiya, Y., Concentration Fluctuations Induced by Thermally-Driven Local Order Changes in the Molten Binary Alloy. *Scandinavian Journal of Metallurgy* **2001**, *30*, 345-351.
88. Kurita, R.; Murata, K.; Tanaka, H., Control of Fluidity and Miscibility of a Binary Liquid Mixture by the Liquid-Liquid Transition. *Nat Mater* **2008**, *7*, 647-652.
89. Dudowicz, J.; Douglas, J. F.; Freed, K. F., Self-Assembly in a Polymer Matrix and Its Impact on Phase Separation. *J Phys Chem B* **2009**, *113*, 3920-3931.
90. Tsuchiya, Y., The Thermodynamics of Structural Changes in the Liquid Sulphur-Tellurium System: Compressibility and Ehrenfest's Relations. *Journal of Physics-Condensed Matter* **1994**, *6*, 2451-2458.
91. Tsuchiya, Y.; Kakinuma, F.; Bergman, C., Structural Changes and Concentration Fluctuations in the Liquid Se-Te System. *J Non-Cryst Solids* **1996**, *205*, 143-146.
92. Coulet, M. V.; Bergman, C.; Bellissent, R.; Bichara, C., Local Order and Phase Separation in Sulphur-Tellurium Melts: A Neutron Scattering Study. *J Non-Cryst Solids* **1999**, *250*, 463-467.
93. Coulet, M.-V.; Bellissent, R.; Bichara, C., Closed-Loop Miscibility Gap in Sulfur-Tellurium Melts: Structural Evidence and Thermodynamic Modelling. *Journal of Physics-Condensed Matter* **2006**, *18*, 11471-11486.

94. Graf, E. H.; Lee, D. M.; Reppy, J. D., Phase Separation and the Superfluid Transition in Liquid He3-He4 Mixtures. *Phys. Rev. Lett.* **1967**, *19*, 417.
95. Leiderer, P.; Bosch, W., Universality of Tricritical He3-He4 Mixtures under Pressure. *Phys. Rev. Lett.* **1980**, *45*, 727-729.
96. Feynman, R. P., The Gamma-Transition in Liquid Helium. *Phys. Rev.* **1953**, *90*, 1116-1117.
97. Feynman, R. P., Atomic Theory of the 2-Fluid Model of Liquid Helium. *Phys. Rev.* **1954**, *94*, 262-277.
98. Zhang, H.; Zhong, C.; Douglas, J. F.; Wang, X. D.; Cao, Q. P.; Zhang, D. X.; Jiang, J. Z., Role of String-Like Collective Atomic Motion on Diffusion and Structural Relaxation in Glass Forming Cu-Zr Alloys. *Journal of Chemical Physics* **2015**, *142*, 164506.
99. Dudowicz, J.; Freed, K. F.; Douglas, J. F., Lattice Model of Living Polymerization. Ii. Interplay between Polymerization and Phase Stability. *Journal of Chemical Physics* **2000**, *112*, 1002-1010.
100. Zhou, Z.; Chu, B., Phase Behavior and Association Properties of Poly(Oxypropylene)-Poly(Oxyethylene)-Poly(Oxypropylene) Triblock Copolymer in Aqueous Solution. *Macromolecules* **1994**, *27*, 2025-2033.
101. Huff, A.; Patton, K.; Odhner, H.; Jacobs, D. T.; Clover, B. C.; Greer, S. C., Micellization and Phase Separation for Triblock Copolymer 17r4 in H2o and in D2o. *Langmuir* **2011**, *27*, 1707-1712.
102. Tan, H. M.; Moet, A.; Hiltner, A.; Baer, E., Thermoreversible Gelation of Atactic Polystyrene Solutions. *Macromolecules* **1983**, *16*, 28-34.
103. Kawanishi, K.; Takeda, Y.; Inoue, T., The Sol-Gel Transition and the Liquid-Liquid Phase Separation in Poly(Vinyl Chloride) Solutions. *Polym. J.* **1986**, *18*, 411-416.
104. Tanaka, H., Liquid–Liquid Transition and Polyamorphism. *Journal of Chemical Physics* **2020**, *153*, 130901.
105. Rah, K.; Freed, K. F.; Dudowicz, J.; Douglas, J. F., Lattice Model of Equilibrium Polymerization. V. Scattering Properties and the Width of the Critical Regime for Phase Separation. *Journal of Chemical Physics* **2006**, *124*, 144906.
106. Kumar, P.; Buldyrev, S. V.; Becker, S. R.; Poole, P. H.; Starr, F. W.; Stanley, H. E., Relation between the Widom Line and the Breakdown of the Stokes-Einstein Relation in Supercooled Water. *P Natl Acad Sci USA* **2007**, *104*, 9575-9579.
107. Simeoni, G. G.; Bryk, T.; Gorelli, F. A.; Krisch, M.; Ruocco, G.; Santoro, M.; Scopigno, T., The Widom Line as the Crossover between Liquid-Like and Gas-Like Behaviour in Supercritical Fluids. *Nature Physics, in press* **2010**, *6*, 503-507.
108. Xu, L. M.; Kumar, P.; Buldyrev, S. V.; Chen, S. H.; Poole, P. H.; Sciortino, F.; Stanley, H. E., Relation between the Widom Line and the Dynamic Crossover in Systems with a Liquid-Liquid Phase Transition. *P Natl Acad Sci USA* **2005**, *102*, 16558-16562.
109. Dudowicz, J.; Freed, K. F.; Douglas, J. F., Flory-Huggins Model of Equilibrium Polymerization and Phase Separation in the Stockmayer Fluid. *Phys. Rev. Lett.* **2004**, *92*, 045502.
110. Van Workum, K.; Douglas, J. F., Equilibrium Polymerization in the Stockmayer Fluid as a Model of Supermolecular Self-Organization. *Phys. Rev. E* **2005**, *71*, 031502.
111. Nishikawa, K.; Tanaka, I., Correlation Lengths and Density Fluctuations in Supercritical States of Carbon Dioxide. *Chemical Physics Letters* **1995**, *244*, 149-152.
112. Nishikawa, K.; Morita, T., Fluid Behavior at Supercritical States Studied by Small-Angle X-Ray Scattering. *J. Supercrit. Fluids* **1998**, *13*, 143-148.
113. Nishikawa, K.; Kusano, K.; Arai, A. A.; Morita, T., Density Fluctuation of a Van Der Waals Fluid in Supercritical State. *Journal of Chemical Physics* **2003**, *118*, 1341-1346.
114. Arai, A. A.; Morita, T.; Nishikawa, K., Analysis to Obtain Precise Density Fluctuation of Supercritical Fluids by Small-Angle X-Ray Scattering. *Chemical Physics* **2005**, *310*, 123-128.
115. Zhu, J. L.; Zhang, P. W.; Wang, H.; Delle Site, L., Is There a Third Order Phase Transition for Supercritical Fluids? *Journal of Chemical Physics* **2014**, *140*, 014502.
116. Angell, C. A., The Amorphous State Equivalent of Crystallization: New Glass Types by First Order Transition from Liquids, Crystals, and Biopolymers. *Solid State Sci.* **2000**, *2*, 791-805.

117. Angell, C. A.; Moynihan, C. T.; Hemmati, M., 'Strong' and 'Superstrong' Liquids, and an Approach to the Perfect Glass State Via Phase Transition. *J Non-Cryst Solids* **2000**, *274*, 319-331.
118. Torquato, S., Hyperuniform States of Matter. *Phys Rep* **2018**, *745*, 1-95.
119. Xu, W. S.; Douglas, J. F.; Freed, K. F., Influence of Cohesive Energy on the Thermodynamic Properties of a Model Glass-Forming Polymer Melt. *Macromolecules* **2016**, *49*, 8341-8354.
120. Angell, C. A., Two-State Thermodynamics and Transport Properties for Water from "Bond Lattice" Model. *Journal of Physical Chemistry* **1971**, *75*, 3698-&.
121. Moynihan, C. T.; Angell, C. A., Bond Lattice or Excitation Model Analysis of the Configurational Entropy of Molecular Liquids. *J Non-Cryst Solids* **2000**, *274*, 131-138.
122. Zhang, H.; Wang, X. Y.; Chremos, A.; Douglas, J. F., Superionic Uo2: A Model Anharmonic Crystalline Material. *Journal of Chemical Physics* **2019**, *150*, 174506.
